# Over a decade of atmospheric mercury monitoring at Amsterdam Island in the French Southern and Antarctic Lands

**DOI:** 10.1038/s41597-023-02740-9

**Published:** 2023-11-28

**Authors:** Olivier Magand, Hélène Angot, Yann Bertrand, Jeroen E. Sonke, Laure Laffont, Solène Duperray, Léa Collignon, Damien Boulanger, Aurélien Dommergue

**Affiliations:** 1 Observatoire des Sciences de l’Univers à La Réunion (OSU-R), UAR 3365, CNRS, Université de La Réunion, Météo France, 97744 Saint-Denis, La Réunion France; 2 Univ. Grenoble Alpes, CNRS, INRAE, IRD, Grenoble INP, IGE, Grenoble, France; 3 Géosciences Environnement Toulouse, CNRS/IRD, Université Paul Sabatier Toulouse 3, Toulouse, France; 4 CNRS, Observatoire Midi-Pyrénées, SEDOO, Toulouse, France

**Keywords:** Atmospheric chemistry, Environmental monitoring

## Abstract

The Minamata Convention, a global and legally binding treaty that entered into force in 2017, aims to protect human health and the environment from harmful mercury (Hg) effects by reducing anthropogenic Hg emissions and environmental levels. The Conference of the Parties is to periodically evaluate the Convention’s effectiveness, starting in 2023, using existing monitoring data and observed trends. Monitoring atmospheric Hg levels has been proposed as a key indicator. However, data gaps exist, especially in the Southern Hemisphere. Here, we present over a decade of atmospheric Hg monitoring data at Amsterdam Island (37.80°S, 77.55°E), in the remote southern Indian Ocean. Datasets include gaseous elemental and oxidised Hg species ambient air concentrations from either active/continuous or passive/discrete acquisition methods, and annual total Hg wet deposition fluxes. These datasets are made available to the community to support policy-making and further scientific advancements.

## Background & Summary

Mercury (Hg) is a ubiquitous toxicant harmful to human health and the environment^1^. This global contamination issue is addressed under the 2017 Minamata Convention (https://www.mercuryconvention.org/en) which commits its current 147 parties to curb anthropogenic Hg emissions to air and releases to land and water. According to Article 22 of the Convention, the Conference of the Parties (COP) is required to periodically evaluate the effectiveness of the Convention starting in 2023, and to perform this evaluation on the basis of available scientific information. The overarching goal of the effectiveness evaluation is to assess whether actions taken under the umbrella of the Minamata Convention have resulted in changes in Hg levels in the environment. Monitoring of atmospheric Hg levels and associated trend analysis has been identified as one of the primary and most appropriate tools to help evaluate the Convention’s effectiveness^2^. While Hg cycles through all environmental reservoirs, the atmosphere responds to changes in emissions much more quickly (within months) than terrestrial and oceanic reservoirs (years to decades)^3,4^.

**Fig. 1:**
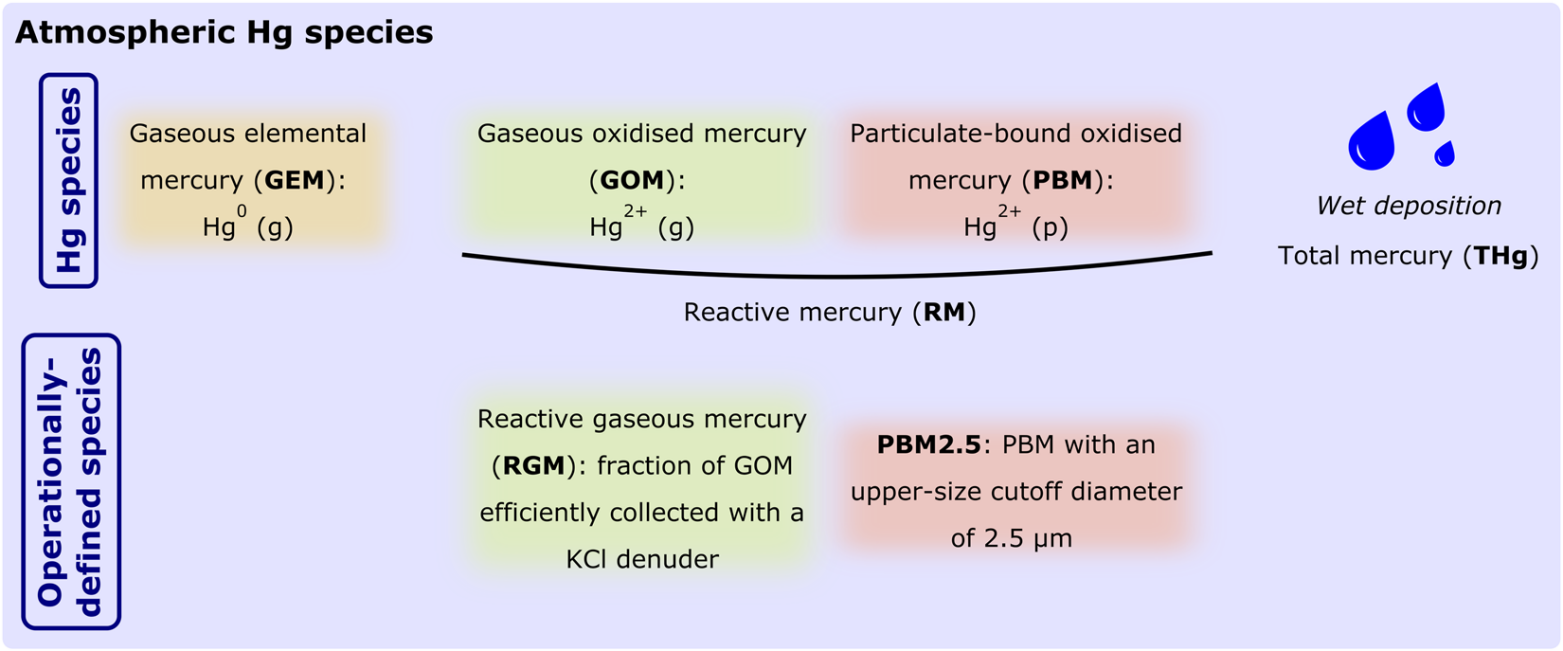
Atmospheric Hg species monitored at Amsterdam Island. These acronyms are those commonly used in the Hg community and are summarised here to facilitate the reader.

Hg exists in three forms in the atmosphere (Fig. 1): gaseous elemental mercury (GEM), the dominant form of atmospheric Hg, and two oxidised forms, gaseous oxidised mercury (GOM) and particulate-bound mercury (PBM). These three Hg species can be deposited to ecosystems through wet and dry processes. In the guidance report UNEP/MC/COP.4/INF/12 on monitoring Hg and Hg compounds to support the effectiveness evaluation of the Minamata Convention^5^, a three-tier approach is recommended, with a gradual increase in complexity. Tier 1 focuses on GEM and wet deposition monitoring through automated, manual, or passive sampling, and on the collection of ancillary meteorological variables. Tiers 2 and 3 involve advanced techniques for atmospheric Hg measurements (e.g., dry deposition, Hg isotope measurements) and ancillary data (e.g., carbon monoxide, ozone, particulate matter measurements). Given the analytical challenges e.g.^6^, GOM and PBM are currently not recommended for monitoring in Tier 1. However, as noted in the guidance report UNEP/MC/COP.4/INF/12, “several monitoring networks and research groups perform Hg speciation measurements in a comparable manner and are encouraged to share these results, as their data will be helpful in answering questions for the effectiveness evaluation.” Further scientific work will improve understanding of biases in existing methods and comparability across measurement techniques.

**Fig. 2:**
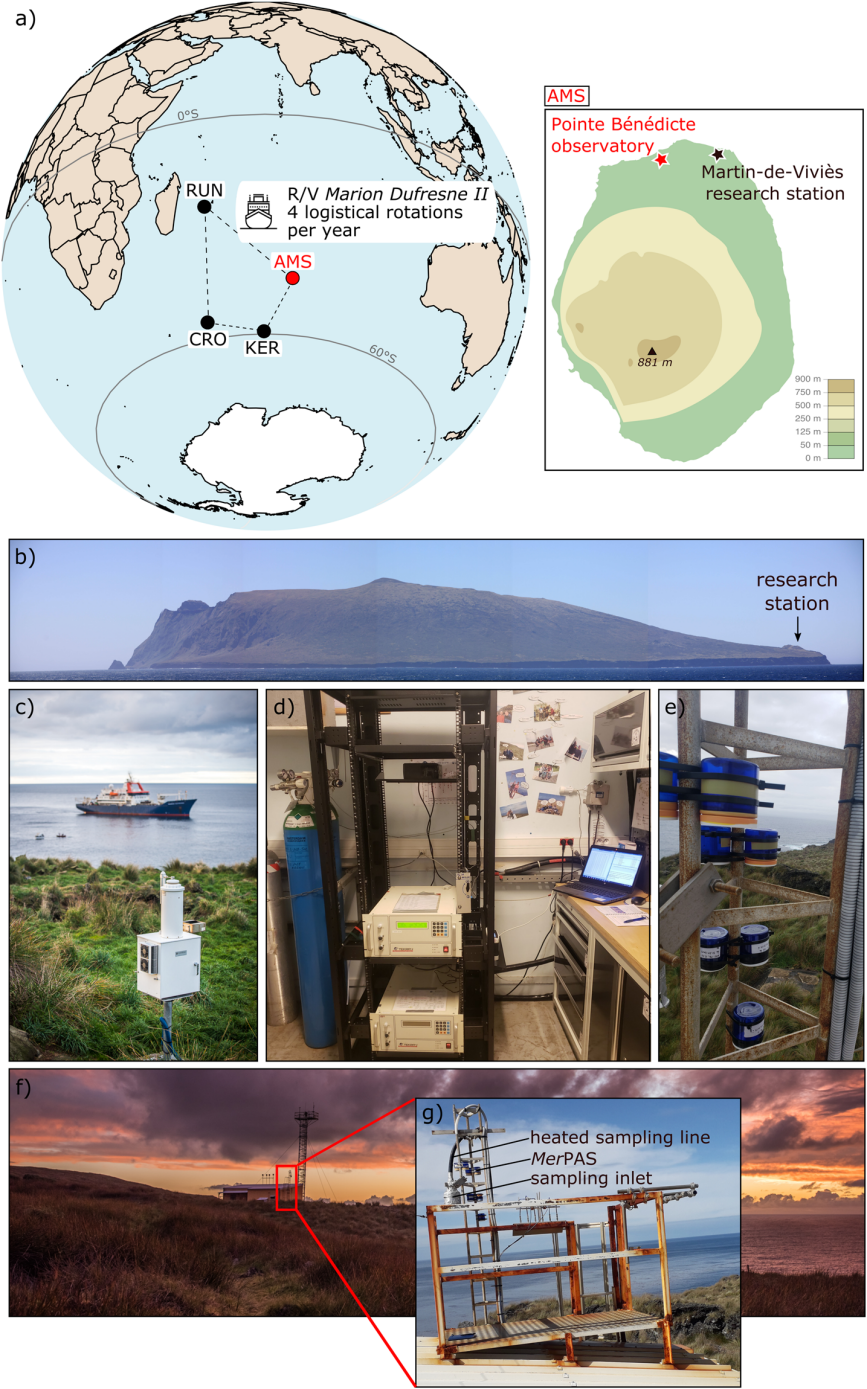
Site location. (**a**) Location of Amsterdam Island (AMS) in the southern Indian Ocean. The island, only inhabited with approximately 20 overwintering crew members, is supplied four times a year by RV *Marion Dufresne II* (in April, August, November, and December) departing from Reunion Island (RUN). The ship also resupplies research stations located on Crozet (CRO) and Kerguelen (KER) islands, also part of the French Southern and Antarctic Lands. Most of the scientific instrumentation is located at the Pointe Bénédicte observatory, 2 km upwind from the Martin-de-Viviès main research station. (**b**) Panoramic view of the island. (**c**) Wet only collector with RV *Marion Dufresne II* in the background. (**d**) Interior of the Pointe Bénédicte observatory with two Tekran instruments for active/continuous measurements of GEM. (**e**) *Mer*PAS systems for passive/discrete measurements of GEM. (**f**) Panoramic view of the Pointe Bénédicte observatory. (**g**) Rooftop sampling platform for atmospheric mercury measurements.

Atmospheric Hg has been successfully monitored for decades through dedicated regional and global networks but with clear data gaps identified in the Southern Hemisphere^7,8^. This issue of data coverage is particularly problematic given the natural and anthropogenic differences between hemispheres that affect the Hg biogeochemical cycle^8,9^ and, ultimately, the effectiveness evaluation. Here, we give an overview of atmospheric Hg monitoring activities carried out at Amsterdam Island (AMS; 37.80°S, 77.55°E; Fig. 2) in the southern Indian Ocean since 2012^7,10^. Being one of the world’s most remote islands, AMS is the ideal location for monitoring the Southern Hemisphere atmospheric background. Monitoring activities have been carried out there for more than 40 years, including monitoring of greenhouse gases and other pollutants^10–23^. The site is currently labelled global GAW/WMO (Global Atmospheric Watch/World Meteorological Organisation) and hosts monitoring activities that are part of international initiatives such as the Integrated Carbon Observation System (ICOS; https://www.icos-cp.eu) and, since 2012, the Global Observation System for Mercury (GOS^4^M; http://www.gos4m.org/)^7,10,12,13,22^.

In an effort to support the effectiveness evaluation of the Minamata Convention, we report here Hg datasets recommended in Tier 1, i.e., ambient air GEM concentrations and total mercury (THg) wet deposition fluxes. Our datasets of oxidised Hg species (referred to as Reactive Mercury; see Fig. 1) are also being shared with the community to promote scientific progress and better understanding of the Hg cycle.

Early subsets of these observations have already been described in the literature, as detailed below: (1) For GEM active/continuous measurements, the period covered was 2012 to 2017^7,10,12,13^, with no subsequent publication available for datasets collected from 2018 onwards. (2) For GEM passive/discrete measurements, the range was from November 2018 to November 2021^24^, with no further description available for datasets collected since then. (3) In terms of RM active/continuous measurements, data from 2012 and 2013 were previously discussed^10^; however, there is no additional description of datasets collected afterwards. (4) As for RM active/discrete measurements, no description of this dataset has been presented up to the present date. (5) Lastly, wet deposition fluxes from 2013 to 2019 were published^22,25^, without additional details provided for datasets collected from 2020 onwards.

In addition to presenting unpublished datasets collected in recent years, this data descriptor provides the first comprehensive overview of all Hg measurements performed at this site since 2012. It also offers a detailed description of all changes in instrumental setup since 2012 that may affect trend analysis, particularly in the context of the effectiveness evaluation of the Minamata Convention.

## Methods

### Study area

AMS is located halfway between South Africa and Australia (3200 km away from Australia, 2880 km from Reunion Island, 4200 km from South Africa, and 3300 km from the Antarctic coast) (Fig. 2). Emerging from the ocean 700 kyr before present, this small island (about 9.2 km long and 7.4 km wide; 55 km^2^ surface area) is located at the northern margin of the southwest wind zone characterised by prevailing westerly and north-westerly winds with an average speed over 7 m/s^11^. The island is mostly influenced by marine air masses, with occasional airflow from continental regions (Africa and South America) in the late austral winter and early spring (August to November), concomitant with the intense biomass burning season over the African continent^10,12–14,26^.

Most of the atmospheric Hg monitoring activities described in this article are carried out at the Pointe Bénédicte observatory, located at 70 m above sea level and 2 km upwind from the main research station (Fig. 2). THg wet deposition monitoring is carried out elsewhere, at ~30 m above sea level and in the vicinity of Martin-de-Viviès.

### Atmospheric Hg monitoring activities

**Fig. 3:**
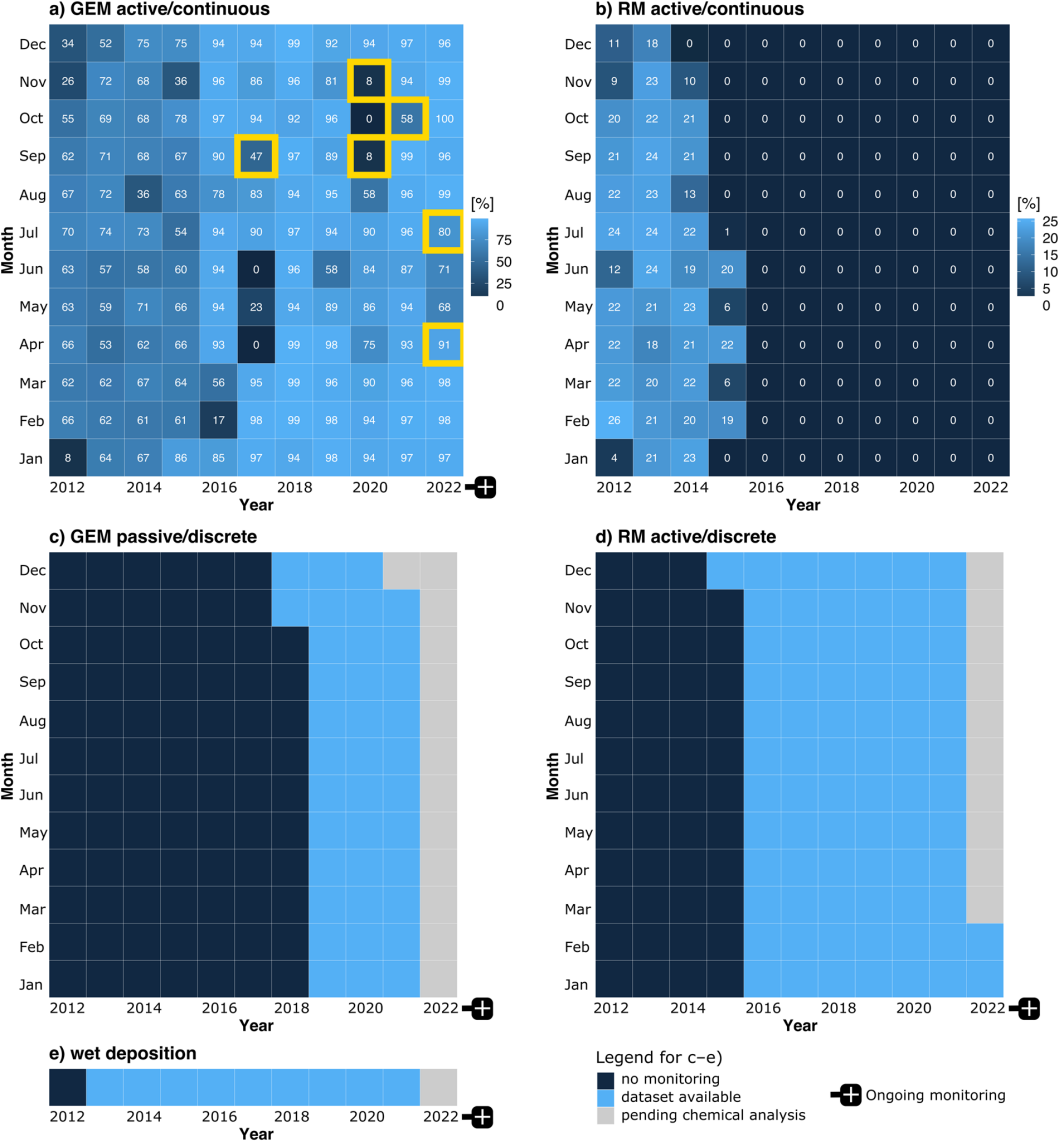
Data coverage. Fraction of valid hourly measurements per month (in %) for (**a**) GEM and (**b**) RM active/continuous monitoring. That fraction is capped at 75% and 25% for GEM and RM, respectively, from Jan 2012 to Nov 2015 due to the operating principle of the Tekran® speciation unit. The yellow rectangles indicate when a new Tekran® 2537 A/B model Hg analyser was installed due to instrument failure. Panels (**c**–**e**) show current data availability for GEM passive/discrete, RM active/discrete, and wet deposition monitoring.

*In situ* Hg monitoring activities are summarised in Fig. 3 and Table 1. THg wet deposition monitoring and active/continuous measurements of GEM and of the operationally-defined RGM and PBM2.5 species (Fig. 1) were initially performed under the framework of the Global Mercury Observation System (GMOS) programme (2011–2015; https://www.gmos.eu/; last access: 13/03/2023). The RM discrete monitoring (2015–present) is now part of the 2016–2025 GEO-flagship Global Observation System for Mercury (https://www.earthobservations.org/, http://www.gos4m.org/; last access: 13/03/2023). Passive GEM measurements (2019–present) were first done in collaboration with the research team that developed the *Mer*PAS system at the University of Toronto^27–30^ and will be fully incorporated in the Canadian-led Global Atmospheric Passive Sampling (GAPS) network in 2024. All these monitoring activities have been implemented at AMS since 2012 within the framework of the GMOStral programme funded by the French Polar Institute.

**Table 1:** List of Hg measurements performed at Amsterdam Island since 2012. See Fig. 1 for the list of acronyms and Fig. 2 for the sampling locations.

Mercury species	Monitoring type	Time period	Time resolution	Sampling location	Instruments or collection system
GEM	active continuous	since Jan 2012 (ongoing)	5 min from Jan 2012 to Nov 2015; 15 min since Nov 2015	Pointe Bénédicte observatory	Cold Vapour Atomic Fluorescence Spectrometer (Tekran® 2537 A/B models)
	passive discrete	since Nov 2019 (ongoing)	Monthly to quarterly	Pointe Bénédicte observatory	Sulphur-impregnated activated carbon sorbent in Passive Air Sampler (Tekran® *Mer*PAS)
RGM and PBM2.5	active continuous	Jan 2012 to Nov 2015	4 hours	Pointe Bénédicte observatory	Speciation unit + Cold Vapour Atomic Fluorescence Spectrometer (Tekran® 1130/1135 + 2537 A/B models)
RM	active discrete	since Nov 2015 (ongoing)	Weekly to monthly	Pointe Bénédicte observatory	Polyethersulfone Cation Exchange Membrane (Millipore®)
THg wet deposition flux	passive discrete	since Mar 2013 (ongoing)	Bi-weekly to monthly	Martin-de-Viviès research station	Automatic wet only collector (Eigenbrodt® NSA-171 model)

#### Gaseous elemental mercury (GEM)

##### Active measurements

A commercial Tekran® 2537 A/B model Hg analyser, commonly used at monitoring sites all over the world^7,31,32^, has continuously been deployed at the Pointe Bénédicte observatory since January 2012^10,12,13^ (Fig. 3a). The operating principle is based on Hg enrichment on dual pure gold cartridges, followed by a thermal desorption and detection by cold vapour atomic fluorescence spectroscopy (CVAFS) (λ = 253.7 nm)^33,34^. Switching between two gold cartridges allows for alternating sampling and desorption modes, and results in continuous measurements. GEM was measured at a time resolution of 5 min from January 2012 to November 2015, and of 15 min since (Table 1). The integration of the signal was optimised in order to avoid potential biases and to allow comparability of the measurements regardless of the sampling frequency (5 vs 15 min), in compliance with international standards^35,36^. This is further discussed in the ***Technical Validation*** section. Ambient air is sampled at 1.2 L per minute through a 10 m long heated (50 °C) and UV protected polytetrafluoroethylene (PTFE) sampling line, with an inlet installed outside at 6 m above ground level (Fig. 2f,g). From January 2012 to November 2015, the instrument was operated in speciation unit mode (see ***reactive mercury species*** section) ensuring that only GEM (as opposed to total gaseous mercury (TGM = GEM + GOM)) was sampled and analysed. Since the uninstallation of the speciation unit in November 2015, we have used two 0.45 µm polyethersulfone cation-exchange membranes (PES-CEM, 0.45 μm, 47 mm, Merck Millipore®) and one 0.45 µm PTFE filter (47 mm diameter), respectively installed at the inlet of the heated line and at the entrance of the instrument. This specific setup prevents any introduction of oxidised species^37^ ensuring that, again, only GEM is sampled and analysed. The instrument is automatically calibrated every 69 h using an internal Hg permeation source which, in turn, is quarterly checked by manual injections of saturated Hg vapour collected from a temperature-controlled Tekran® 2505 Hg vapour calibration unit^38^. The internal mass flow metre controlling the sampling flow rate is also fortnightly checked by a standardised external calibrator to prevent any drift. Concentrations are expressed in nanograms per cubic metre at standard temperature and pressure (STP; 273.15 K, 1013.25 hPa) with an instrumental detection limit below 0.1 ng/m^3^ and a GEM average systematic uncertainty around 10%^12^. The Tekran® 2537 A/B model Hg analyser is operated according to standard operating procedures routinely applied by the Global Mercury Observation System (GMOS), the Canadian Atmospheric Mercury Measurement Network (CAMNet), and the United States Atmospheric Mercury Network (AMNet)^7,39^.

##### Passive measurements

GEM has also been simultaneously measured by passive air samplers (Tekran® *Mer*PAS) at the Pointe Bénédicte observatory since November 2019 (Fig. 3c). These passive samplers have been extensively used and tested under a wide range of climatic conditions^27,28,40–46^, including at AMS^24^, and have been shown to have a precision and accuracy that is comparable to that of state-of-the-art active measurement techniques^28^. They provide an inexpensive and easy-to-use alternative to active measurements and are increasingly used worldwide. GEM is sequestered in a sulphur-impregnated activated carbon sorbent (HGR carbon, Calgon®) cartridge through a collection system using a Radiello diffusive barrier. At AMS, the *Mer*PAS systems (samples, blanks) are deployed on a quarterly basis. They are carefully stored in well-sealed glass jars and in the dark before and after field deployment to avoid contamination and to lower blanks^24^. Back to the laboratory, samples undergo thermal decomposition and amalgamation, and are analysed by atomic absorption spectroscopy (AMA254 (Leco® Instruments Ltd) or MA3000 (Nippon® Instruments Corporation)) using pure oxygen as carrier gas. The analytical procedure and associated metrology (calibration, blank correction, method detection and quantification limits calculation) are described in Hoang *et al*.^24^ and McLagan *et al*.^29^. Final volumetric air concentrations (in ng per cubic metre) are obtained by dividing the field blank-adjusted amount of Hg in each sampler (in ng) by the product of a temperature and wind-corrected sampling rate (m^3^/day) and the deployment duration in days^24^.

#### Reactive mercury species

##### Continuous measurements

A commercial Tekran® 1130/1135 model speciated Hg analyser was deployed at AMS from January 2012 to November 2015 (see Fig. 3b) for the monitoring of Reactive Gaseous Mercury (RGM; a subset of GOM consisting of all forms of Hg sampled using a KCl-coated denuder^47^; see Fig. 1) and Particulate Bound Mercury (PBM2.5, i.e., PBM with an upper-size cutoff diameter of 2.5 µm; see Fig. 1)^7,10^. This so-called speciation unit system, consisting of both Tekran® 1130/1135 modules, was connected to the Tekran® 2537 A/B analyser for simultaneous GEM monitoring (see ***Gaseous Elemental Mercury*** section) through a 10 m long and heated (50 °C) PTFE sampling line. RGM is sequestered by the Tekran® 1130 module KCl-coated denuder while the fraction of PBM below 2.5 µm (PBM2.5; see Fig. 1) is trapped onto a quartz regenerable filter located within the Tekran® 1135 module^47^. At AMS, the 1130 and 1135 modules were configured to collect RGM and PBM2.5 over a three-hour period at a 10 L/min flow rate. RGM and PBM2.5 were then sequentially thermally desorbed (500 °C for 15 min and 800 °C for 20 min, respectively) into a Hg-free air stream and subsequently analysed as GEM by the Tekran® 2537 A/B analyser. RGM and PBM2.5 concentrations are expressed in picograms per cubic metre under STP conditions with an instrumental detection limit below 0.4 and 0.3 pg/m^3^ for the Tekran® 1130 and 1135 modules, respectively^48^. By analogy with the Tekran® 2537 A/B model, the 1130/1135 modules were operated following well established standard operating procedures^39^.

##### Discrete measurements

Reactive Mercury (RM = GOM + PBM; see Fig. 1) has been collected since December 2015 (Fig. 3d) using the two PES-CEMs installed at the inlet of the Tekran® 2537 A/B model heated sampling line (see ***Gaseous Elemental Mercury*** section). Gustin *et al*.^49,50^ and Dunham-Cheatham *et al*.^51^ have shown that PES-CEMs collect RM quantitatively. Two PES-CEMs are deployed to limit RM losses due to possible breakthrough^37,52^. Previous studies^37,52–54^ have shown the inertness of such membranes to GEM when deployed in an active sampling setting under environmental background conditions (1 to 2 ng/m^3^) guaranteeing no overestimation of RM and underestimation of GEM. At AMS, time-integrated RM samples are collected at a frequency ranging from ~3 to ~37 days (average ~11 days) depending on local environmental conditions. This sampling frequency ensures the collection of sufficient RM mass on the membranes for further chemical analysis while limiting sampling losses. After collection, the two PES-CEMs are stored separately in Petri dishes inside double-zipper bags and kept dark frozen (−20 °C) until repatriation and chemical analysis. In the laboratory, each filter is placed in a PTFE beaker, digested in 16 mL of 2.5% inverse aqua regia and analysed with a Brooks Rand Model III CVAFS detector. The analytical procedure is further described in Marusczak *et al*.^55^ and Koenig *et al*.^56^. The instrumental method detection limit is estimated to ~5 pg of Hg^56^. The volume of air sampled on the membranes is extracted from the Tekran® 2537 A/B flow rate and RM concentration is consequently expressed in picograms per cubic metre under STP conditions.

#### Wet deposition fluxes

In order to estimate annual wet deposition fluxes, THg collection in precipitation has been carried out at AMS since March 2013 (Fig. 3e) following well-established international protocoles^22^. Rain events are sampled by a commercial Eigenbrodt® NSA-171/KE automatic wet only collector^22,25^. The start of a rain event induces an impulse from the infrared precipitation sensor and causes the lid to open up as follows: the lid moves up, swings to the side, and sinks down to prevent aerodynamic interference. Precipitation impacting a 100 mm borosilicate-glass funnel flows through a PTFE pipe directly into a 1 L fluorinated high density polyethylene (FLPE) sample bottle containing 0.8% v/v 30% concentrated Suprapur® quality hydrochloric acid. When precipitation stops, a signal from the precipitation sensor causes the collection funnel to close, ensuring that only wet fallout is collected, without interference from dry deposits. Evaporation of volatile Hg is prevented by maintaining a constant indoor temperature (below the outdoor temperature) and by using a vapour lock connected to the sampling bottle. Every single component of the sampling system is composed of chemically neutral material and is carefully cleaned with acid, following the procedure reported in Tassone *et al*.^57^ and adapted from the US-EPA 1631 method^58^. Integrated samples are collected over periods ranging from ~6 to ~45 days, depending on the season and on the occurrence of exceptional rainfall events, with an average fortnightly and monthly collection frequency in wet and dry periods, respectively. Precipitation samples are then kept frozen (−20 °C) and in the dark (to avoid photo-induced reduction of Hg species) until repatriation and further chemical analysis. Field, transport, bottle, and reagent blanks are also regularly collected and analysed^25,57,59^. The complete analytical procedure and associated metrology can be found in Tassone *et al*.^25,57^. THg values are derived according to the UNI 15853:2010 method and converted into volume-weighted mean concentration values. Annual THg wet deposition fluxes are then calculated as reported in Sprovieri *et al*.^22^.

## Data Records

Our datasets are available under Creative Commons Attribution 4.0 International (CC-BY 4.0) Licence from the GMOS-FR AERIS website (https://gmos.aeris-data.fr/ last access: 13/03/2023). As summarised in Table 2, level 1 and 2 data products are available on the AERIS website for active/continuous GEM measurements^60,61^, discrete GEM measurements^62^, RGM/PBM2.5 active/continuous measurements^60^, RM active measurements^63^, and THg wet deposition fluxes^64^. Tables 3–6 summarise the list of attributes for each qualified and downloadable dataset. Level 1 data represent quality-checked datasets in their original time resolution. Level 2 data, when available, are modified quality-checked data products. The GEM level 2 dataset provides hourly averaged GEM concentrations calculated from quality-controlled level 1 GEM data (5- or 15-min time resolution) when the hourly recovery rate exceeds 50% (i.e., number of valid data points vs. that possible over the reporting period). Level 2 annual THg wet deposition fluxes give the annual flux calculated based on the individual rain samples collected during the corresponding year. It is important to note that the periods considered for each annual flux do not always strictly correspond to a calendar year starting on January 1^st^ and ending on December 31^th^ due to logistical constraints and depending on rainfall events. Users can refer to variables ‘Date_time_START’ and ‘Date_time_STOP’ for more information (see Table 6).

**Table 2:** Data records. Level 1 datasets represent quality-checked datasets with data in their original time resolution. Level 2 data, when available, are modified quality-checked data products (hourly mean for GEM; annual wet deposition flux). See Fig. 1 for the list of acronyms.

Mercury species	Frequency	Level	Time resolution	DOI	Reference number
GEM	active continuous	1	5/15 min	Angot, H., Dommergue, A., Magand, O. & Bertrand, Y. (2023). Continuous measurements of atmospheric mercury at Amsterdam Island (L1). [Dataset]. Aeris. 10.25326/345#v1.0	^60^
		2	1 hour	Angot, H., Dommergue, A., Magand, O. & Bertrand, Y. (2023). Continuous measurements of atmospheric mercury at Amsterdam Island (L2). [Dataset]. Aeris. 10.25326/168#v1.0	^61^
	passive discrete	1	Monthly to quarterly	Angot, H., Dommergue, A., Magand, O. & Bertrand, Y. (2023). Discrete measurements of atmospheric elemental mercury at Amsterdam Island (L1). [Dataset]. Aeris. 10.25326/489#v1.0	^62^
RGM PBM2.5	active continuous	1	4 hours	Angot, H., Dommergue, A., Magand, O. & Bertrand, Y. (2023). Continuous measurements of atmospheric mercury at Amsterdam Island (L1). [Dataset]. Aeris. 10.25326/345#v1.0	^60^
RM	active discrete	1	Weekly to monthly	Angot, H., Dommergue, A., Magand, O. & Bertrand, Y. (2023). Discrete measurements of atmospheric reactive mercury at Amsterdam Island (L1). [Dataset]. Aeris. 10.25326/488#v1.0	^63^
THg wet deposition flux	passive discrete	2	Annual	Angot, H., Dommergue, A., Magand, O. & Bertrand, Y. (2023). Total mercury wet deposition fluxes at Amsterdam Island (L2). [Dataset]. Aeris. 10.25326/487#v1.0	^64^

**Table 3:** List of attributes in the files corresponding to active/continuous measurements of GEM, RGM, and PBM2.5 with a Tekran® 2537/1130/1135 Hg analyser. Note that RGM and PBM2.5 datasets are only available from 2012 to 2015.

Mercury species	Qualification level	Variable	Definition
GEM, RGM, PBM2.5	L1	Date_time	Date and time of measurement in local time (UTC + 5; DD/MM/YYYY HH:MM:SS)
		GEM_valid	Ambient air concentration of Gaseous Elemental Mercury (GEM) in ng/m^3^ at standard temperature and pressure (5–15 min time resolution)
		RGM_valid	Ambient air concentration of Reactive Gaseous Mercury (RGM) in pg/m^3^ at standard temperature and pressure (4 hours time resolution)
		PBM_valid	Ambient air concentration of Particulate Bound Mercury with an upper-size cutoff diameter of 2.5 μm (PBM2.5) in pg/m^3^ at standard temperature and pressure (4 hours time resolution)
GEM	L2	Date_time	Date and time of measurement in local time (UTC + 5, DD/MM/YYYY HH:MM:SS)
		GEM_valid	Hourly-averaged ambient air concentration of Gaseous Elemental Mercury (GEM) in ng/m^3^ at standard temperature and pressure

**Table 4:** List of attributes in the files corresponding to passive/discrete measurements of GEM with *Mer*PAS samplers.

Mercury species	Qualification level	Variable	Definition
GEM	L1	# sample	Sample identification number
		Date_time_START	Date and time of collection start in local time (UTC + 5; DD/MM/YYYY HH:MM:SS)
		Date_time_STOP	Date and time of collection finish in local time (UTC + 5; DD/MM/YYYY HH:MM:SS)
		Duration	Duration of sample collection in days
		T_avg	Average ambient air temperature in Celsius degrees during sample collection
		WS_avg	Average wind speed in m/s during sample collection
		SR_adj	Adjusted sampling rate in m^3^/day
		GEM_valid	Ambient air concentration of Gaseous Elemental Mercury (GEM) in ng/m^3^

**Table 5:** List of attributes in the files corresponding to active/discrete measurements of RM with polyethersulfone cation exchange membranes.

Mercury species	Qualification level	Variable	Definition
RM	L1	# sample	Sample identification number
		Date_time_START	Date and time of collection start in local time (UTC + 5; DD/MM/YYYY HH:MM:SS)
		Date_time_STOP	Date and time of collection finish in local time (UTC + 5; DD/MM/YYYY HH:MM:SS)
		Hg_mass	Mass of mercury on the sample in pg
		LOD	Analytical limit of detection in pg
		RM_valid	Ambient air Reactive Mercury (RM) concentration in pg/m^3^ at standard temperature and pressure

**Table 6:** List of attributes in the files corresponding to THg wet deposition measurements.

Mercury species	Qualification level	Variable	Definition
THg wet deposition flux	L2	Year	Calendar year considered
		Date_time_START	Date and time of collection start in local time (UTC + 5; DD/MM/YYYY HH:MM:SS)
		Date_time_STOP	Date and time of collection finish in local time (UTC + 5; DD/MM/YYYY HH:MM:SS)
		Total_collection_days_used	Number of collection days used for flux calculation
		Wet_dep_flux	Annual wet deposition flux in µg/m^2^/year

## Technical Validation

### Active/continuous measurements of gaseous elemental mercury (GEM) and reactive mercury (RGM and PBM2.5)

To ensure the comparability and the quality of the GEM/RGM/PBM2.5 active/continuous measurements, dedicated instruments and all retrieved data are respectively operated and quality controlled following established SOPs routinely applied by monitoring networks such as GMOS, CAMNet, and AMNet^7,39^ (see ***Methods*** section). AMS being a background air monitoring site (i.e., low atmospheric levels corresponding to ~1 ng/m^3^ or less for GEM), we have further optimised the instrumental detection capacities of the Tekran® 2537 A/B Hg analysers. This optimisation process, discussed with and validated by the manufacturer, guarantees the best possible sensitivity for low-level detection and quantification. More specifically, two actions were undertaken: (1) implementation of a new set of peak integration settings to improve quantification at low sample mass loading (Table 7) as discussed in Swartzendruber *et al*.^35^, and (2) increase of the residence time in the detection cuvette by reducing the argon carrier gas flow rate to half of the manufacturer default settings while remaining within the range of recommended values (40 ml/mn and 100 ml/mn in “measure” and “flush flow” instrumental modes vs. 80 and 200 ml/mn in default settings).

**Table 7:** Tekran® 2537 A/B integration parameters optimisation for Hg peak detection in low-level ambient air concentration conditions. ***N-up*** is the number of consecutive up marks required to register an upslope condition; ***V-up*** is the size of each increase when Hg is detected, in analog to digital (A/D) counts (LSBs) required to be qualified as an up mark; ***N-dn*** is the number of consecutive down marks required to register a downslope condition; ***V-dn*** is the size of a decrease, also in A/D counts required to qualify as a down mark; ***NBase*** if the number of consecutive no changes required to register a baseline condition after a downslope has been detected and finally, ***VBase*** corresponds to the permissible change allowed from one reading to the next to qualify as a “no change” condition. Once ***NBase*** consecutive no changes have been registered, the first such reading is considered to be the end of the peak and a baseline condition is consequently flagged. LSB (Least Significant Bit) is the smallest level that an A/D can convert.

**Tekran® default integration settings**	***N-up***: 7	***N-dn***: 3	***NBase***: 5
	***V-up***: 5	***V-dn***: 3 LSB	***VBase***: 8 LSB
**Optimised low-level integration settings**	***N-up***: 6	***N-dn***: 4	***NBase***: 19
	***V-up***: 4	***V-dn***: 3 LSB	***VBase***: 1 LSB

**Fig. 4:**
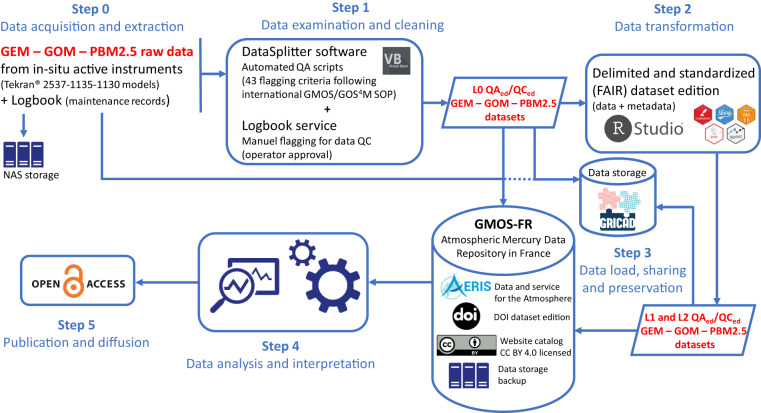
Data processing workflow for GEM, GOM, and PBM2.5 active/continuous measurements (adapted from Magand *et al*.^66^). Only level 1 and 2 datasets are publicly available on the GMOS-FR AERIS website.

Quality control procedures are applied at each step of the data processing chain, from the raw measurement to the provision of the qualified dataset. Standardised quality assurance measures and calibration tools are applied on-site to provide documented and traceable data and data products. Raw datasets as well as routine or exceptional maintenance files are compiled and processed by a custom-built software developed at the Institute of Environmental Geosciences (Grenoble, France) specifically designed for the QA/QC of the GEM/RGM/PBM2.5 datasets. In this automated process, the raw dataset is flagged (valid, warning, invalid) according to 43 possible criteria corresponding to all operation phases of the instrument (e.g., calculation of Hg concentration, calibration, sensitivity of the instrument). The inclusion of all field notes implying further invalidations (e.g., during maintenance operations) allows the production of a fully QA/QC’d dataset. Our data processing procedure is relatively close to the one developed under the umbrella of the GMOS project (G-DQM^65^) but accounts for first-hand inputs from the site manager (e.g., field notes). A detailed description of this QA/QC procedure is available on the French national GMOS-FR AERIS data portal, reported in various publications^7,10,66^ and briefly summarised in Fig. 4.

It should be noted that, since 2012, we have had to replace the Tekran® 2537 instrument 6 times due to instrument failure or unresolved technical issues (Fig. 3a). These replacements were made following strict operating procedures (see above), with a strong focus on calibration tests, to prevent the introduction of systematic bias. Despite these occasional instrumental issues, the fraction of valid hourly measurements per month generally exceeded the 66% minimum WMO GAW requirement for continuous measurements^67^ (72 out of 84 months; see Fig. 3a). From 2012–2015, this minimum requirement can be reduced to 50% given the operating principle of the Tekran® speciation unit (25% of the operating time of the 2537 analyser dedicated to RGM/PBM2.5 measurements). That target was also generally reached (43 out of 48 months; see Fig. 3a).

### Discrete measurements of Gaseous elemental mercury (GEM), reactive mercury (RM), and THg wet deposition

**Fig. 5:**
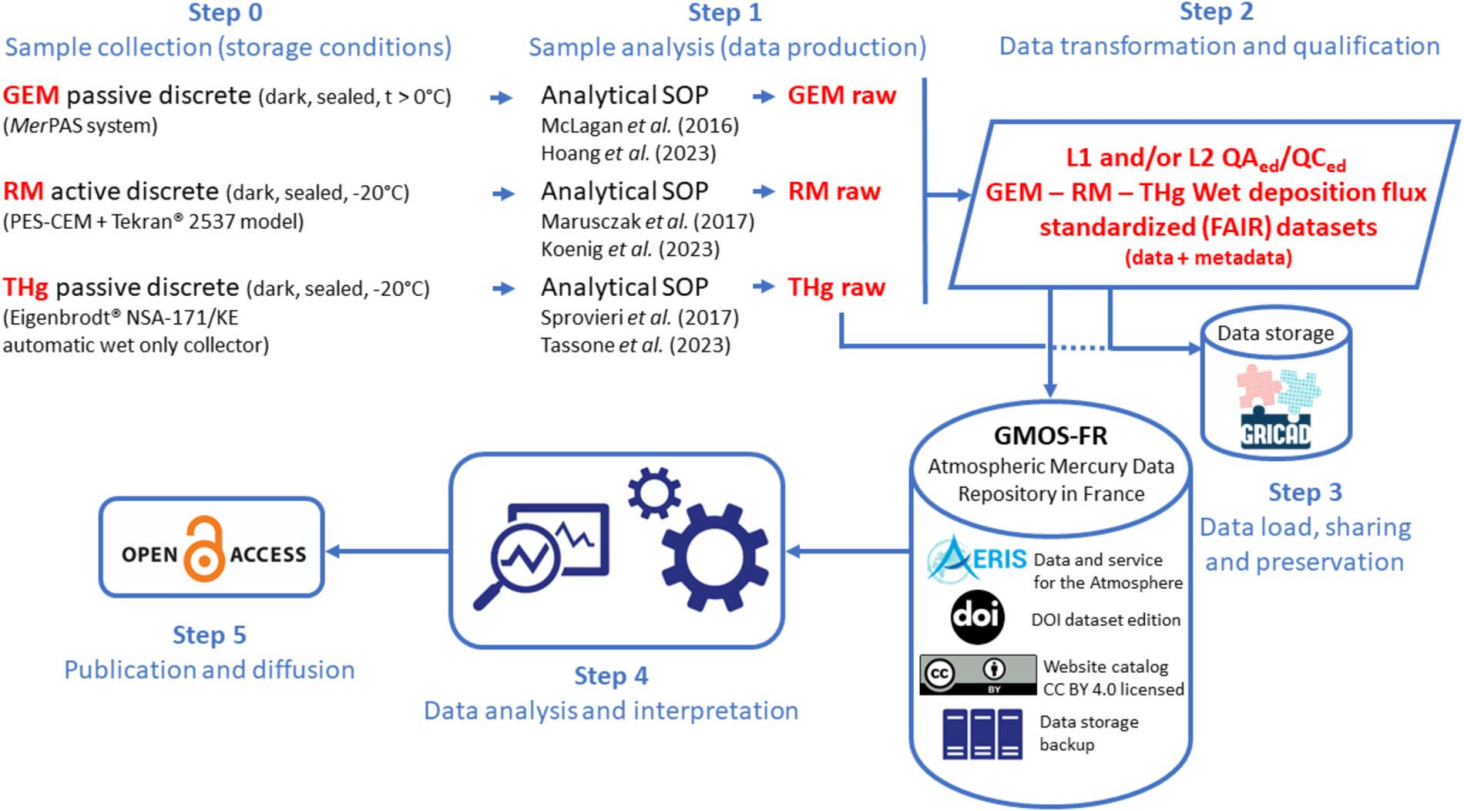
Data processing workflow for discrete GEM, RM, and wet deposition measurements.

Figure 5 synthesises the data processing workflow related to these datasets.

Operating and analytical procedures related to passive measurements of GEM with *Mer*PAS systems are described in McLagan *et al*.^27,28,30^. Additional tests were made to (1) evaluate the dependence of the passive sampling rate on meteorological conditions encountered at AMS and (2) to lower blanks. The results, reported in Hoang *et al*.^24^, highlight the quality of our operating protocols. In addition, we follow the procedure described in McLagan *et al*.^29^ during the subsequent chemical analysis step to prevent sulphur poisoning of catalysts.

**Fig. 6:**
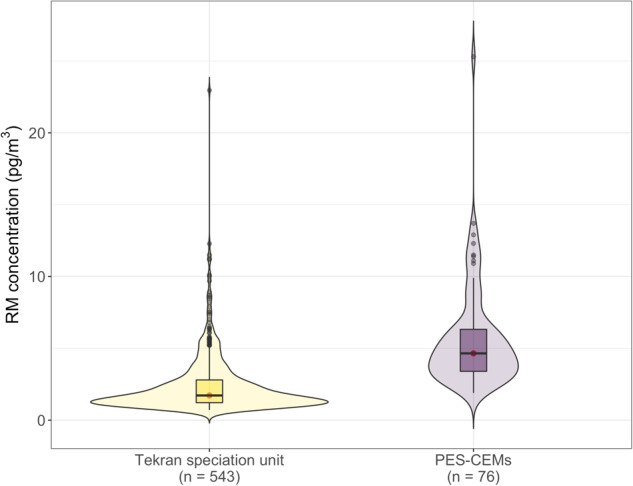
Reactive Mercury (RM) concentrations inferred from the Tekran® speciation unit (from 2012 to 2015; RM = RGM + PBM2.5) and from the use of polyether sulfone cation-exchange membranes (PES-CEMs; from Dec 2015 to Feb 2022). n indicates the number of data points/samples. Note that RM measurements are still ongoing using PES-CEMs but samples collected after Feb 2022 have not been analysed yet (see Fig. 3d). The violin plots show the kernel probability density and include a marker for the median (in red) and a box indicating the interquartile range (IQR). As in standard boxplots, the upper (lower) whisker extends from the box to the largest (smallest) value no further than 1.5 × IQR. Values below the detection limit were discarded.

Since 2015, RM species have been collected on two successive PES-CEMs (0.45 μm, 47 mm, Merck Millipore®) installed at the inlet of the Tekran® 2537 A/B model heated sampling line. The decision to switch from automatic and high frequency RGM/PBM2.5 measurements to discrete RM measurements was made based on the very low concentrations observed over the 2012–2015 period^10^ and on the need to reduce costs and power consumption. This decision was further reinforced by the growing body of literature demonstrating (1) potential sampling biases associated with the Tekran® speciation unit^6,52,53,68–70^ and (2) the very good performances of PES-CEMs^50,51,55^. Depending on the speciation of oxidised Hg (e.g., HgCl_2_, HgBr_2_, HgO, Hg(NO_3_)_2_, HgSO_4_), the collection efficiency of PES-CEMs is indeed 1.3 to 12 times higher than that of the Tekran® speciation unit^6,50,52^. Figure 6 shows the distribution of RM concentrations observed at AMS with the Tekran® speciation unit (RGM + PBM2.5) and with PES-CEMs and confirms that RM concentrations inferred from the Tekran® speciation unit at AMS are slightly biased low by a factor of 2.7 (median (interquartile range): 1.7 (1.6) vs. 4.7 (2.9) pg/m^3^), in line with the literature.

Operating and analytical procedures related to the determination of the THg wet deposition flux are described in Sprovieri *et al*.^22^ and Tassone *et al*.^25^ and follow the GMOS SOP adapted from the US EPA method 1631E^57^. QA/QC procedures include duplicate sample analysis, precision testing using a certified reference material, matrix spikes, and regular system, transport, reagent, and field blank analysis^25^.

## Usage Notes

The standardised *.csv file format permits easy import into all analysis software commonly used in the atmospheric science community. The datasets can be used without further processing. In addition to datasets and associated metadata, the GMOS-FR AERIS website also includes a list of peer-reviewed publications that can help better understand the current state of science associated with these datasets. User should be aware that the RGM/PBM2.5 datasets collected with a Tekran® speciation unit may be biased, as discussed above. It is essential to consider these biases when interpreting and utilizing the data.

The data presented in this manuscript have undergone peer review in 2023 and are represented by the specific versions (#v1.0) given in Table 2. As monitoring activities are still ongoing, new datasets will be regularly uploaded to the GMOS-FR AERIS data portal. These new versions might include additional information (e.g., additional monitoring years) or refined data. Please note that any updates or new versions of the datasets are not part of the peer-reviewed data associated with this manuscript.

We welcome enquiries regarding ancillary datasets also collected at AMS by partners (e.g., meteorological conditions, greenhouse gases or ozone ambient air mole fractions) that could help interpret atmospheric Hg time-series.

## Data Availability

No custom code has been used during the generation of these datasets.
